# Constructing of *Bacillus subtilis*-Based Lux-Biosensors with the Use of Stress-Inducible Promoters

**DOI:** 10.3390/ijms22179571

**Published:** 2021-09-03

**Authors:** Andrew G. Kessenikh, Uliana S. Novoyatlova, Sergey V. Bazhenov, Eugeniya A. Stepanova, Svetlana A. Khrulnova, Eugeny Yu. Gnuchikh, Vera Yu. Kotova, Anna A. Kudryavtseva, Maxim V. Bermeshev, Ilya V. Manukhov

**Affiliations:** 1 Research Center for Molecular Mechanisms of Aging and Age-Related Diseases, Moscow Institute of Physics and Technology, 141701 Dolgoprudny, Russia; kessenikh.ag@phystech.edu (A.G.K.); novoyatlova.us@phystech.edu (U.S.N.); bazhenov1994@gmail.com (S.V.B.); stepanovaevgenya111@mail.ru (E.A.S.); khrulnovas@mail.ru (S.A.K.); kudryavtseva@phystech.edu (A.A.K.); 2 Faculty of Physics, HSE University, 109028 Moscow, Russia; 3 Department of Clinical Bacteriology, Mycology, and Antibiotic Treatment, National Research Center for Hematology, 125167 Moscow, Russia; 4 Kurchatov Genomic Center, State Research Institute of Genetics and Selection of Industrial Microorganisms of the National Research Centre «Kurchatov Institute», 117545 Moscow, Russia; gnuchih_evgeniy@mail.ru; 5 National Research Center «Kurchatov Institute», Kurchatov Complex for Genetic Research, 123098 Moscow, Russia; v-kotova@mail.ru; 6 State Reseach Institute of Genetics and Selection of Industrial Microorganisms of the National Research Centre «Kurchatov Institute», 117545 Moscow, Russia; 7 Federal Research Center of Biological Systems and Agro-Technologies of RAS, 460000 Orenburg, Russia; 8 Topchiev Institute of Petrochemical Synthesis, Russian Academy of Sciences, 119071 Moscow, Russia; bmv@ips.ac.ru

**Keywords:** bacterial biosensor, *Bacillus subtilis*, genotoxicity assessment, soil analysis, oxidative stress, inducible promoters

## Abstract

Here, we present a new lux-biosensor based on *Bacillus subtilis* for detecting of DNA-tropic and oxidative stress-causing agents. Hybrid plasmids pNK-DinC, pNK-AlkA, and pNK-MrgA have been constructed, in which the *Photorhabdus luminescens* reporter genes *luxABCDE* are transcribed from the stress-inducible promoters of *B. subtilis*: the SOS promoter P*dinC*, the methylation-specific response promoter P*alkA*, and the oxidative stress promoter P*mrgA*. The luminescence of *B. subtilis-*based biosensors specifically increases in response to the appearance in the environment of such common toxicants as mitomycin C, methyl methanesulfonate, and H_2_O_2_. Comparison with *Escherichia coli*-based lux-biosensors, where the promoters P*dinI*, P*alkA*, and P*dps* were used, showed generally similar characteristics. However, for *B. subtilis* P*dinC*, a higher response amplitude was observed, and for *B. subtilis* P*alkA*, on the contrary, both the amplitude and the range of detectable toxicant concentrations were decreased. *B. subtilis* P*dinC* and *B. subtilis* P*mrgA* showed increased sensitivity to the genotoxic effects of the 2,2′-bis(bicyclo [2.2.1] heptane) compound, which is a promising propellant, compared to *E. coli*-based lux-biosensors. The obtained biosensors are applicable for detection of toxicants introduced into soil. Such bacillary biosensors can be used to study the differences in the mechanisms of toxicity against Gram-positive and Gram-negative bacteria.

## 1. Introduction

For testing chemical impurities (toxins) and other biologically active substances, primarily medications, luminescent biosensor cells (lux-biosensors) are currently used in two ways: (1) based on bioluminescence quenching [1,2,3], and (2) based on bioluminescence induction [4,5,6,7,8,9,10,11,12,13]. The existing lux-biosensors based on genetically engineered *Escherichia coli* cells and a number of naturally luminescent enterobacteria are of little use for working with soil pollution [14]. The design of lux-biosensors based on Gram-positive *Bacillus subtilis* bacteria could contribute to the analysis of the mechanisms of toxicity of medicinal products that specifically interact with Gram-positive bacteria, or the ecological monitoring of soil contamination.

The construction of a *lux* operon that is efficiently expressed in Gram-positive bacteria is complicated due to the need to replace the Shine–Dalgarno sequence upstream of each reading frame. Previously, such works were carried out and luminescent bacteria of *Streptococcus*, *Staphylococcus*, *Bacillus,* and a number of other genera were obtained, which were used mainly for medical applications [2,15,16]. The luminescence of these bacteria was constitutive, and it was used to determine the integral effect of biologically active substances on bacterial cells.

Promoters with the highest response amplitude are considered more promising for the development of lux-biosensors. According to works [17,18] in the SOS-regulon of *B. subtilis*, the *dinC* gene promoter has the highest response amplitude—36–175 folds. The activation of DNA glycosylase promoters in response to DNA alkylation in *B. subtilis* cells is not so high and for P*alkA* it is only 2.5 times, which was determined by the changes in the released 7-methylguanine after treatment with nitrosoguanidine [19]. *E. coli* has two regulons specifically activated by the superoxide anion radical (SoxR/S) and hydrogen peroxide (OxyR/S) [20,21,22]. There is not such a clear division of the response under oxidative stress in *B. subtilis*; genes that respond to oxidative stress can be activated by both hydrogen peroxide and paraquat [23]. The greatest increase in transcription is observed for promoters of *katA* and *mrgA*—the genes encoding catalase and ferritin-like DNA-binding protein, respectively [23,24,25,26].

In this work, we present whole-cell lux-biosensors based on *B. subtilis* cells, constructed using stress-inducible promoters: P*dinC*, P*alkA*, and P*mrgA*.

## 2. Results

### 2.1. Characterization of the B. subtilis 168 pNK-DinC Lux-Biosensor for SOS-Response Detection

**Figure 1:**
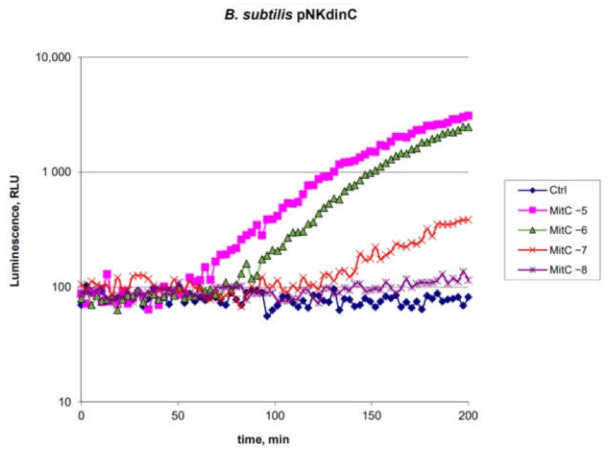
Time dependence of the luminescence of the *B. subtilis* 168 pNK-DinC cell culture supplemented with MitC. “Ctrl”—biosensor cells without the addition of toxicant; “MitC −5”—biosensor cells with 10 μM MitC added; “MitC −6”—1 μM; “MitC −7”—100 nM; and “MitC −8”—10 nM, correspondently.

The characteristics of *B. subtilis* 168 pNK-DinC were investigated using the antibiotic mitomycin C (MitC), a well-known drug inducing the SOS-response in *E. coli* cells [27]. The cells were preliminarily grown to OD = 0.4 at 37 °C and divided into 200 µL portions. After MitC was added to final concentrations of 10 μM, 1 μM, 100 nM, and 10 nM, cells were incubated at room temperature for 3 h with periodic luminescence measurements. The kinetic curves on Figure 1 are the results of individual experiments, results of which are representative and consistent with the other experiments.

The data shown in Figure 1 indicate that the designed *B. subtilis* 168 pNK-DinC lux-biosensor is induced by MitC and, therefore, is sensitive to DNA damage. The threshold concentration of MitC is about 10 nM, which generally corresponds to the sensitivity to MitC of the best *E. coli*-based lux-biosensors for determining the SOS response, in which the P*colD* promoter of the *cda* gene from the conjugative plasmid pColD-CA23 [22] or the P*dinI* promoter [28] are fused with *luxCDABE*. The time of the onset of biosensor activation is about 1 h with a maximum activation after 3 h. It is approximately two times slower than that for *E. coli*-based biosensor cells. The maximum response amplitude exceeds one order of magnitude and is 40-fold for 10 μM MitC.

### 2.2. Characterization of the B. subtilis 168 pNK-MrgA Lux-Biosensor for Oxidative Stress Detection

**Figure 2:**
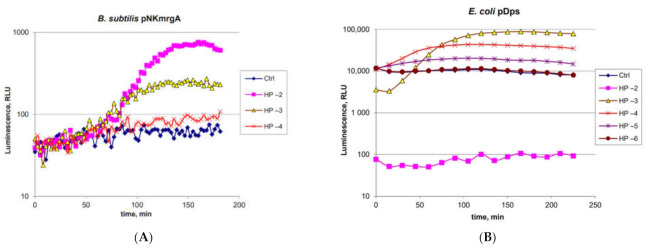
Time dependence of the luminescence of *B. subtilis* 168 pNK-MrgA (**A**) and *E. coli* MG1655 pDps (**B**) cells induced by hydrogen peroxide in different concentrations. “Ctrl”—biosensor cells without the addition of toxicant; “HP −2”—biosensor cells with 10 mM H_2_O_2_ added; “HP −3”—1 mM; “HP −4”—100 µM; “HP −5”—10 µM; “HP −6”—1 µM.

The functionality of *B. subtilis* 168 pNK-MrgA was tested by adding hydrogen peroxide to the biosensor cell culture to final concentrations of 10 mM, 1 mM, and 100 μM (Figure 2A). In *E. coli* cells, the homologue of the *mrgA* gene is *dps* [29]; therefore, we compared the characteristics of these biosensors. We used the *E. coli* MG1655 pDps biosensor with the same H_2_O_2_ concentrations (Figure 2B). The *B. subtilis* and *E. coli* cell cultures were preliminarily grown at 37 °C to OD = 0.4 and 0.1, respectively, and divided into portions, which were supplemented with H_2_O_2_ in different concentrations.

From the data in Figure 2A, it can be seen that the P*mrgA* promoter in *B. subtilis* cells is inducible by H_2_O_2_. Biosensor induction begins 1 h after H_2_O_2_ addition and reaches its maximum amplitude in 2.5 h. The threshold concentration of H_2_O_2_ for this biosensor is 100 μM. The maximum response amplitude exceeds an order of magnitude and is 14 times for an H_2_O_2_ concentration of 10 mM. The biosensor *E. coli* MG1655 pDps is characterized by high sensitivity to H_2_O_2_ and the rather pronounced toxic effect of it at high concentrations (10 mM and 1 mM of H_2_O_2_); immediately after the addition of the toxicant (“0” point at time axis), the biosensor’s luminescence drops by two orders of magnitude and three times, respectively (Figure 2B).

*B. subtilis* 168 pNK-MrgA shows about an order of magnitude reduced sensitivity to H_2_O_2_ compared with *E. coli* MG1655 pDps (Figure 2B). However, the width of working concentrations range for both biosensors is the same and is more than two orders of magnitude: from 100 μM to 10 mM for *B. subtilis* 168 pNK-MrgA and from 10 μM to 1 mM for *E. coli* MG1655 pDps. The response time of the *E. coli*-based biosensor is shorter—its induction begins in approximately 15 min and reaches the maximum in around 2.5 h. Thus, the optimal measurement time for both biosensors is about 2.5–3 h. The amplitude of the response for biosensors *B. subtilis* 168 pNK-MrgA and *E. coli* MG1655 pDps is approximately the same—10–15 times.

### 2.3. Characterization of the B. subtilis 168 pNK-AlkA Lux-Biosensor for DNA Alkylation Detection

**Figure 3:**
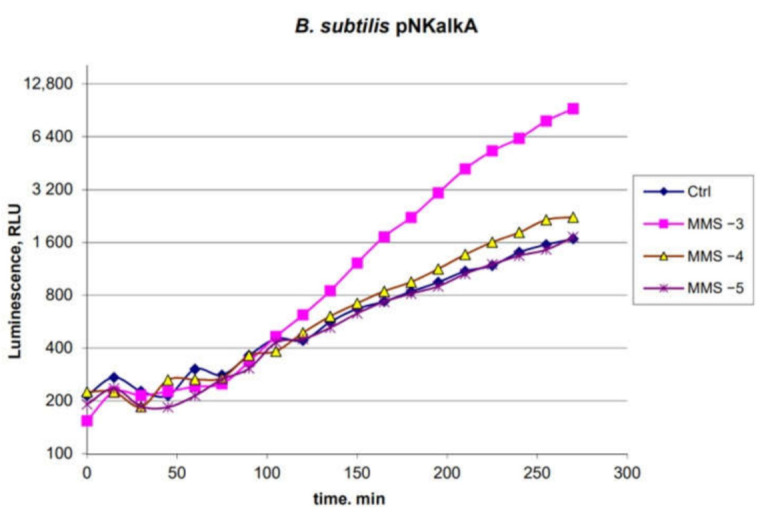
Time dependence of the luminescence of the *B. subtilis* 168 pNK-AlkA cell culture after the addition of MMS. “Ctrl”—biosensor cells without the addition of toxicant; “MMS −3”—biosensor cells with 1 mM MMS added; “MMS −4”—100 μM; “MMS −5”—10 µM.

The characteristics of the *B. subtilis* 168 pNK-AlkA biosensor were studied using the known alkylating agent methyl methanesulfonate (MMS) (Figure 3), which activates the *alkA* gene promoter in *E. coli* [30]. MMS was used at final concentrations of 1 mM, 100 µM, and 10 µM—the measurement time was prolonged to 4.5 h to obtain a higher response amplitude.

As can be seen from the data in Figure 3, *B. subtilis* 168 pNK-AlkA is inducible by MMS. The threshold concentration is about 100 μM. The response time of the biosensor is about 2 h, with a maximum activation after 4 h. The maximum response amplitude does not exceed an order of magnitude and is 6 times for the MMS concentration of 1 mM. The *B. subtilis* 168 pNK-AlkA has the same sensitivity as the *E. coli*-based biosensor with P*alkA* transcriptionally fused with the β-galactosidase gene according to data from [30]. However, lux-biosensors that use luciferase as a reporter are usually more sensitive. This could be illustrated by comparing the sensitivity of biosensors from [11,31] and [30] to MNNG (N-methyl-N’-nitro-N-nitrosoguanidine)—threshold concentrations of MNNG were 10 nM for the *lux*-biosensor and 100 nM for the β-galactosidase-based biosensor.

### 2.4. Main Characteristics of the Obtained Biosensors

Data on the main characteristics of the obtained biosensors, namely, sensitivity, induction amplitude, dynamic range (working concentrations range) and response time are represented in Table 1.

One can see from Table 1 that *B. subtilis*-based biosensors have comparable characteristics with biosensors based on *E. coli* cells.

Biosensors *E. coli* pAlkA-lux and *B. subtilis* pNK-AlkA have strong specificity and are inducible only by alkylating agents, such as MMS, without cross-induction by other toxicants. Biosensors *E. coli* pDps and *B. subtilis* pNK-MrgA are inducible only by H_2_O_2_. For biosensors *E. coli* pDinI and *B. subtilis* pNK-DinC, the cross-induction by non-specific toxicants was observed. These biosensors are also inducible by H_2_O_2_ and MMS at high concentrations. This is quite expected because large-scale oxidative damage can stop the replication fork and induce the SOS response [31]. Multiple alkylation of nucleotide bases with drugs such as MMS can also lead to the appearance of extended single-stranded regions during repair and induce the SOS response [9].

### 2.5. Application of the Obtained Lux-Biosensors for Assessment of BBH Toxicity against Gram-Positive Bacteria

**Figure 4:**
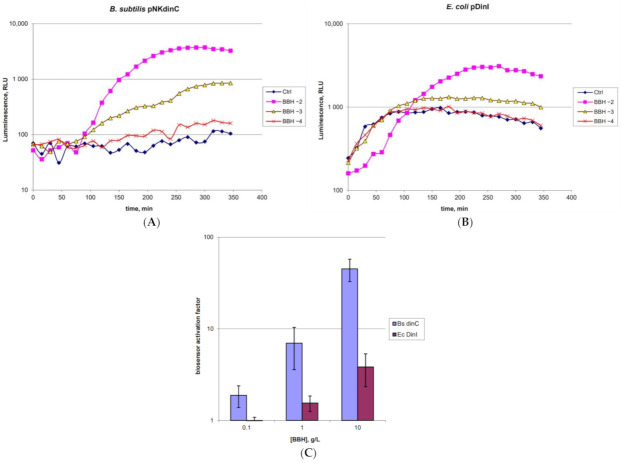
Time dependence of the luminescence of *B. subtilis* 168 pNK-DinC (**A**) and *E. coli* MG1655 pDinI (**B**) biosensors after adding BBH. The comparison of luminescence induction coefficients of these biosensors in 4 h after adding BBH, taken from the results of 5 experiments (**C**). “Bs dinC”—*B. subtilis* 168 pNK-DinC; “Ec DinI”—*E. coli* MG1655 pDinI; “Ctrl”—biosensor cells without added toxicants, “BBH −2”, “BBH −3”, and “BBH −4”—biosensor cells with BBH added at concentrations of 10, 1, and 0.1 g/L, correspondently.

**Figure 5:**
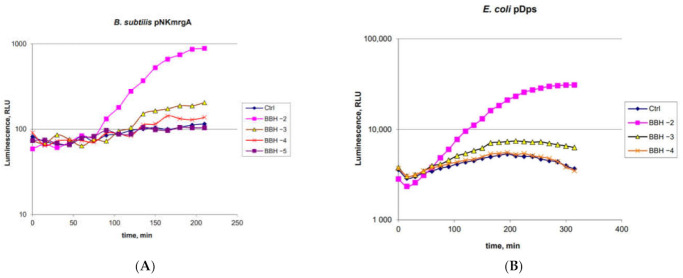
Time dependence of the luminescence of *B. subtilis* 168 pNK-MrgA (**A**) and *E. coli* MG1655 pDps (**B**) biosensors after BBH addition. “Ctrl”—biosensor cells without addition of toxicant; “BBH −2”, “BBH −3”, “BBH −4”, and “BBH −5”—biosensor cells with BBH added at concentrations of 10, 1, 0.1, and 0.01 g/L, correspondently.

The toxic effect of BBH (2,2′-bis(bicyclo[2.2.1] heptane), a promising compound as a propellant, on *B. subtilis* cells was investigated using biosensors *B. subtilis* 168 pNK-DinC and *B. subtilis* 168 pNK-MrgA (Figure 4A and Figure 5A). For comparison, *E. coli* MG1655 pDps and *E. coli* MG1655 pDinI lux-biosensors were used (Figure 4B,C and Figure 5B). The measurements were conducted with freshly grown cell cultures of OD = 0.4 for *B. subtilis* and OD = 0.1 for *E. coli*. After adding BBH to final concentrations of 10, 1, and 0.1 g/L, the cells were incubated at room temperature for 4 h with periodic luminescence measurements (Figure 4).

According to the data obtained, the threshold concentration of BBH for *B. subtilis* 168 pNK-DinC is 0.1 g/L. For the *E. coli* MG1655 pDinI biosensor—1 g/L, the same as that for *E. coli* MG1655 pColD-lux biosensor, according the work [32]. The maximum response amplitude for *B. subtilis* 168 pNK-DinC was 50-fold, while for *E. coli* pDinI, it was only 4 (Figure 4C). The time of the beginning of biosensors induction by BBH is approximately the same for *E. coli* and *B. subtilis*—about 1.5 h.

A pair of biosensors, *B. subtilis* 168 pNK-MrgA and *E. coli* pDps, was used to determine the ability of BBH to induce oxidative stress (Figure 5).

The threshold BBH concentration for *B. subtilis* 168 pNK-MrgA is between 0.1 and 1 g/L (Figure 5A). This is a higher sensitivity compared to *E. coli* pDps (1 g/L) (Figure 5B) and the *E. coli* pOxyR-lux biosensors (specific to H_2_O_2_, according to [32], its sensitivity to BBH is 1 g/L), and it is almost equal to the sensitivity of the *E. coli* pSoxS-lux biosensor, which is specific to the superoxide anion radical. The maximum response amplitude for both *B. subtilis* 168 pNK-MrgA and *E. coli* pDps was about 10 times. The initiation time of biosensor induction is approximately the same—about 1 h.

### 2.6. Application of the B. subtilis 168 pNKdinC Lux-Biosensor for Detection of Toxicants Introduced into the Soils

**Figure 6:**
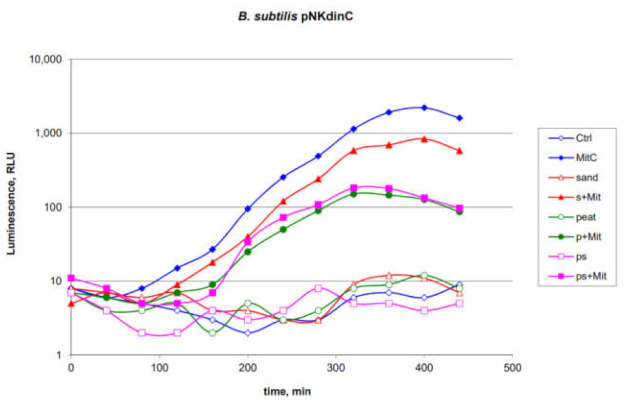
Time dependence of the luminescence of *B. subtilis* 168 pNK-DinC biosensor cell cultures after adding MitC directly to the medium, soil, and soil supplemented with MitC. “Ctrl”—biosensor cells without the addition of toxicant; “MitC”—100 nM MitC added; “sand”—biosensor cells with sandy soil added; “peat”—peat soil; “ps”—sandy-peat soil; “s + Mit”—biosensor cells with soil, which was preliminary supplemented with MitC (final concentration of MitC in medium with biosensor cells and soil-mix was 100 nM); “p + Mit”—peat with MitC; and “ps + Mit”—peat-sand soil with MitC.

*B. subtilis* 168 pNK-DinC was chosen to test the efficiency of biosensors in the field when the soil samples contaminated with toxicants are tested. In these experiments, MitC was added to various soils of the Arctic region collected on the coast of the Kandalaksha Bay of the White Sea: sandy, peaty and mixed. Thirty-milligram soil samples with or without MitC were added to the 200 µL portions of biosensor cell culture. The final concentration of MitC in the medium with biosensor cell culture was 100 nM. As a positive control, MitC was directly added to the biosensor cell culture to a final concentration of 100 nM. Then, the samples were incubated at 16–17 °C with periodic measurement of luminescence (Figure 6).

As can be seen from the data shown in Figure 6, the studied soils themselves do not possess genotoxic properties. The addition of sandy soil leads to an approximately 2-fold decrease in the response amplitude of the biosensor to MitC. Soils containing peat decrease the response amplitude by about 10 times. Such a decrease in the response can be explained by the partial shielding of luminescence by dark soil particles and partial binding of the toxicant. Soil acidity also plays a significant role, while peat bogs in Karelia are characterized by low pH [33], which could significantly decrease the characteristics of *E. coli*-based biosensors [14].

## 3. Discussion

Whole-cell biosensors are widely used for assessing compounds’ toxicity and ecological control [34,35]. Gram-positive and Gram-negative bacteria can have different sensitivities to the same toxicant. Here, we present a new lux-biosensor based on *B. subtilis* for detecting DNA-damaging and oxidative-stress-causing agents. Characteristics of the obtained biosensor strains *B. subtilis* 168 pNK-DinC, *B. subtilis* 168 pNK-AlkA, and *B. subtilis* 168 pNK-MrgA were determined in experiments with the addition of standard toxicants, causing the SOS response, DNA alkylation, and oxidative stress, respectively (Figure 1, Figure 2 and Figure 3). *B. subtilis*-based biosensors showed comparable characteristics with biosensors based on *E. coli* cells. New Gram-positive biosensors complement the bacterial test-systems, which consist of well-proven *E. coli*-based lux-biosensors [4,11,22,36].

It is known from the works [32,37] that strained hydrocarbon compounds are capable of causing DNA damage and oxidative stress, but not DNA alkylation in living cells. In *E. coli* cells, there are no homologues to the *dinC* gene of *B. subtilis*; therefore, the lux-biosensor *E. coli* pDinI, related to the SOS regulon and having a relatively high induction amplitude [28], was taken for comparison. Studies of the toxic properties of BBH have shown (Figure 4 and Figure 5) that the biosensors *B. subtilis* 168 pNK-DinC and *B. subtilis* 168 pNK-MrgA have a higher sensitivity and response amplitude compared to analogous biosensors based on *E. coli* cells. This can be explained by the difference in permeability of the cell wall of Gram-positive and Gram-negative cells by compounds of the BBH type. It should be noted that there is no homologue of the *E. coli* SoxS/SoxR type regulation system in *B. subtilis*, which specifically reacts to the appearance of the superoxide anion radical [23]. This may increase the toxicity of BBH to *B. subtilis* compared to *E. coli* and serve as another explanation for the effect obtained. Consequently, the sensitivity of the *mrgA* gene promoter appears to be higher than that of P*dps* (Figure 5).

Previously, it has been shown that *E. coli*-based biosensors are not suitable for testing acidic peaty soils due to a catastrophic decrease in the ability of cells to luminescence and activation of stress promoters [14]. In contrast, the *B. subtilis* 168 pNK-DinC biosensor strain can be used for direct studies of the genotoxicity of soil samples adjusted to light shielding by soil particles (Figure 6).

## 4. Materials and Methods

### 4.1. Strains and Plasmids

Bacterial strains and plasmids used in the current work are presented in Table 2. *E. coli* MC1061 cells were used for constructing biosensor plasmids for *B. subtilis* cells. *E. coli* MG1655 cells were used for transformation by pDps and pDinI plasmids and obtaining *E. coli*-based biosensors.

### 4.2. Enzymes and DNA Manipulation

Plasmid DNA was isolated by the GeneJET Plasmid Miniprep Kit (ThermoFisher Scientific, Waltham, MA, USA). The *E. coli* cell transformation with hybrid plasmids, agarose gel electrophoresis, and isolation of plasmid and total DNA was performed according to [40]. Restriction was carried out using the SacI restriction enzyme (Promega, Madison, WI, USA). Ligation was conducted with the use of Gibson Assembly, prepared according to [41]. *B. subtilis* cells were transformed according to [42,43].

### 4.3. Chemicals

Enzymes for Gibson Assembly preparation were purchased in NEB (Ipswich, MA, USA). Media were from Helicon (Moscow, Russia). Oligonucleotides were made by Syntol (Moscow, Russia). All chemicals were of analytical purity. Hydrogen peroxide (H_2_O_2_) was obtained from Ferraine (Moscow, Russia). Mitomycin C (MitC) and methyl methanesulfonate (MMS) were obtained from Sigma-Aldrich (St. Louis, MO, USA). 2,2′-bis(bicyclo[2.2.1] heptane) (BBH) was synthetized as in previous work [32]. All test solutions and their dilutions were prepared immediately before use.

### 4.4. Constructing of Biosensor Plasmids

The vector plasmid pPL_ABCDExen (see the plasmid map in Figure S1) was linearized by the SacI restriction enzyme. P*mrgA*, P*alkA*, and P*dinC* promoter regions were amplified by PCR on a matrix of *B. subtilis* 168 genome DNA. Primers, which were used for amplification of promoters and sequencing of insertion in the pPL_ABCDExen vector, are given in Table S1 (Supplementary Materials). Ligation of promoter-containing DNA fragments with linearized vector was conducted with the use of Gibson Assembly. Resulting plasmids, containing promoters of *B. subtilis mrgA*, *alkA*, and *dinC* genes, were named pNK-MrgA, pNK-AlkA, and pNK-DinC, correspondently. For initial transformation and plasmid DNA isolation, *E. coli* MC1061 was used. For sequencing of inserts in the resulting biosensor plasmids, promrev and promdir primers were used (see Table S1). Sequences of promoter regions inserted to obtain pNK-MrgA, pNK-AlkA, and pNK-DinC are given in the supplementary file. Isolated from *E. coli* cells, pNK-MrgA, pNK-AlkA, and pNK-DinC plasmids were used for transformation of *B. subtilis* 168 cells for obtaining biosensor strains.

### 4.5. Culture Medium and Growth Conditions

*E. coli* cell cultures were grown in Lysogeny Broth (LB); *B. subtilis* cells were grown at 37 °C in Brain Heart Infusion (BHI) supplemented with 50 mg/L tryptophan. When measuring luminescence, *B. subtilis* cells were grown with an additional 1% glycerol in the medium. The LB medium was composed of 1% tryptone, 0.5% yeast extract, and 1% NaCl. For obtaining solid media, agar was added to a final concentration of 1.5%. The media were supplemented with 100 μg/mL ampicillin or 10 μg/mL chloramphenicol. For culturing of *B. subtilis*-based biosensor strains, preferably, chloramphenicol was used.

For experiments on determining the sensitivity of biosensors, overnight cultures were used to inoculate liquid LB or BHI to an optical density (OD) of 0.01; the resulting cultures were grown with continuous agitation at 37 °C to the final OD of approximately 0.1 for *E. coli* and 0.4 for *B. subtilis*. The OD of cell suspensions was measured with a KFK-3 photometer (ZOMP, Moscow, Russia).

### 4.6. Measurement of Bioluminescence

Fresh cultures of biosensor strains were divided into 200 μL portions and supplemented with 2 μL common toxicants (MMS, H_2_O_2_, MitC) or BBH in different dilutions. For experiments with soil, samples were prepared in the following way: 30 mg soil samples with or without MitC were added to the 200 µL portions of biosensor cell culture. The final concentration of MitC in the medium with biosensor cell culture was 100 nM. As a positive control, MitC was directly added to the biosensor cell culture to a final concentration of 100 nM.

Bioluminescence intensity of 200 μL portions of cell culture was measured in the 96-well plates using the SynergyHT (Biotek Instruments, Winooski, VT, USA) equipment, or in capless microtubes using Biotox-7BM (BioPhysTech, Moscow, Russia), which is 100 times more sensitive. Luminescence values were expressed in relative light units (RLU), specific to each luminometer. Cell culture portions were incubated at room temperature for 3 to 5 h with periodical bioluminescence measurements. Longevity of incubation with measurements was determined by the average response time for every lux-biosensor individually.

### 4.7. Data Processing

All experiments were conducted in 5 replications. Kinetic curves on graphs were the results of individual experiments, results of which are consistent with the others in a row. For calculations of the main characteristics of biosensors’ maximum induction coefficients, induction start time, etc., average values and standard deviations were calculated for five independent replications. The biosensor induction factor was calculated by dividing the luminescence of induced cells by the luminescence of control cells.

## 5. Conclusions

**Figure 7:**
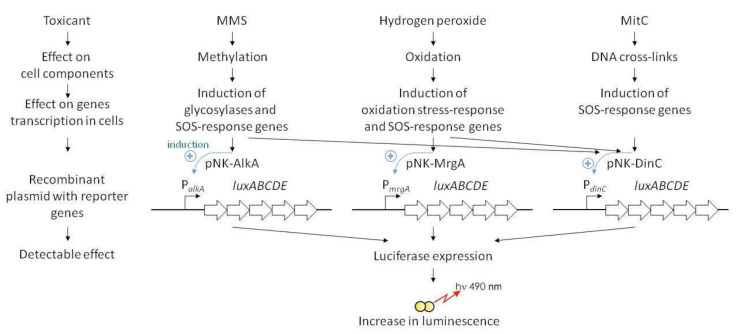
Scheme of working principles of constructed *B. subtilis*-based stress-inducible *lux*-biosensors. On plasmid schemes, clue elements are given: promoters P*alkA*, P*mrgA*, and P*dinC* are from *B. subtilis*, the *luxABCDE* genes are from *P. luminescens*.

In general, results of the investigations demonstrate the operability of the constructed *B. subtilis*-based biosensors (Figure 7).

Tests with standard toxicants causing the SOS response, DNA alkylation and oxidative stress showed comparable characteristics of biosensors based on *B. subtilis* cells with those based on *E. coli* cells. An important advantage of *B. subtilis*-based biosensors is their applicability for direct measurements of the toxicity of polluted soils. Lux-biosensors constructed in this work can complement the *E. coli*-based test system created earlier. The combination of *E. coli*-based biosensors with newly constructed *B. subtilis* ones can be used to study the mechanisms of toxicity of medicines and pollutants that could have different effects on Gram-positive and Gram-negative bacteria.

## Figures and Tables

**Table 1:** Matching the main characteristics of *E. coli*- and *B. subtilis*-based *lux*-biosensors.

Toxicant		Biosensor	*E. coli* pAlkA-Lux	*B. subtilis* pNK-AlkA	*E. coli* pDps	*B. subtilis* pNK-MrgA	*E. coli* pDinI	*B. subtilis* pNK-DinC
	Characteristic							
MitC	Threshold concentration (LOD), M		n/a	n/a	n/a	n/a	(5 ± 3) × 10^−9^	(1 ± 0.4) × 10^−8^
	Induction amplitude		n/a	n/a	n/a	n/a	21 ± 9	45 ± 16
	Dynamic range, log_10_ (C_max_/C_min_)		n/a	n/a	n/a	n/a	4 ± 0.3	4 ± 0.3
	Induction start time, min		n/a	n/a	n/a	n/a	29 ± 8	60 ± 20
H_2_O_2_	Threshold concentration (LOD), M		n/a	n/a	(1 ± 0.5) × 10^−5^	(1.3 ± 0.7) × 10^−4^	(3.0 ± 1.6) × 10^−4^	(3.4 ± 2.7) × 10^−4^
	Induction amplitude		n/a	n/a	15 ± 4	14 ± 3	8 ± 5	4.2 ± 2.5
	Dynamic range, log_10_ (C_max_/C_min_)		n/a	n/a	2.3 ± 0.3	2.3 ± 0.6	1.2 ± 0.3	1.2 ± 0.3
	Induction start time, min		n/a	n/a	15 ± 5	60 ± 12	18 ± 7	64 ± 19
MMS	Threshold concentration (LOD), M		(5 ± 3) × 10^−4^	(1.1 ± 0.5) × 10^−4^	n/a	n/a	(2.0 ± 1.2) × 10^−4^	(1.5 ± 0.6) × 10^−4^
	Induction amplitude		12 ± 7	8 ± 4	n/a	n/a	3.2 ± 0.8	2.5 ± 1.0
	Dynamic range, log_10_ (C_max_/C_min_)		2.0 ± 0.3	1.2 ± 0.4	n/a	n/a	1.5 ± 0.3	2.2 ± 0.4
	Induction start time, min		60 ± 15	108 ± 24	n/a	n/a	82 ± 20	95 ± 24

n/a―“not applicable”, toxicant does not induce the luminescence of biosensor cells at any concentrations.

**Table 2:** Plasmids and bacterial strains used in the study.

Name	Description	Source
Bacterial strains		
*E. coli* K12 MC1061	F–D(araA-leu)7697 [araD139]B/r ∆(codB-lacI)3 galK16 galE15(GalS) λ–e14- mcrA0 relA1 rpsL150 spoT1 mcrB1 hsdR2	VKPM (Moscow, Russia)
*E. coli* K12 MG1655	F- ilvG rfb-50 rph-1	VKPM (Moscow, Russia)
*B. subtilis* 168	trpC2	VKPM (Moscow, Russia)
Plasmids		
pPL_ABCDExen	Promoterless shuttle vector with the *luxABCDE* genes from *Photorhabdus luminescens*. The order of genes in the *lux-*operon and RBS upstream of each gene are optimized for *B. subtilis* expression. Two replication origins (from pMW118 and pBS72). Resistance to trimethoprim (Tp^r^), chloramphenicol (Cm^r^), and ampicillin (Ap^r^).	[38]
pNK-AlkA	pPL_ABCDExen vector with insertion of the *B. subtilis* P*alkA* promoter; P*alkA* is transcriptionally fused to *luxCDABE P. luminescens*	This study
pNK-DinC	pPL_ABCDExen vector with insertion of the *B. subtilis* P*alkA* promoter; P*dinC* is transcriptionally fused to *luxCDABE P. luminescens*	This study
pNK-MrgA	pPL_ABCDExen vector with insertion of the *B. subtilis* P*alkA* promoter; P*mrgA* is transcriptionally fused to *luxCDABE P. luminescens*	This study
pDps	The *E. coli* P*dps* promoter was cloned into pDEW201 [5] vector and transcriptionally fused to the reporter genes *luxCDABE P. luminescens.* Ap^r^	[39]
pDinI	The *E. coli* P*dinI* promoter was cloned into pDEW201 [5] vector and transcriptionally fused to the reporter genes *luxCDABE P. luminescens.* Ap^r^	[28]

## Data Availability

All data are available as Supplementary Files or at the GoogleDrive folder https://drive.google.com/drive/folders/10p9cYCrQ6o3JaeTDAUCUvqJIgoO5X3Ub (accessed on 1 September 2021).

## Supplementary Materials

The following are available online at https://www.mdpi.com/article/10.3390/ijms22179571/s1.
