# Supplementary material for: Constructing of Bacillus subtilis-Based Lux-Biosensors with the Use of Stress-Inducible Promoters
Source: Int J Mol Sci. 2021 Sep 3;22(17):9571. doi: 10.3390/ijms22179571 (PMC8431380; doi:10.3390/ijms22179571)
Supplement: Supplementary file 1 [file ijms-22-09571-s001.zip › ijms-1342734-supplementary.pdf]

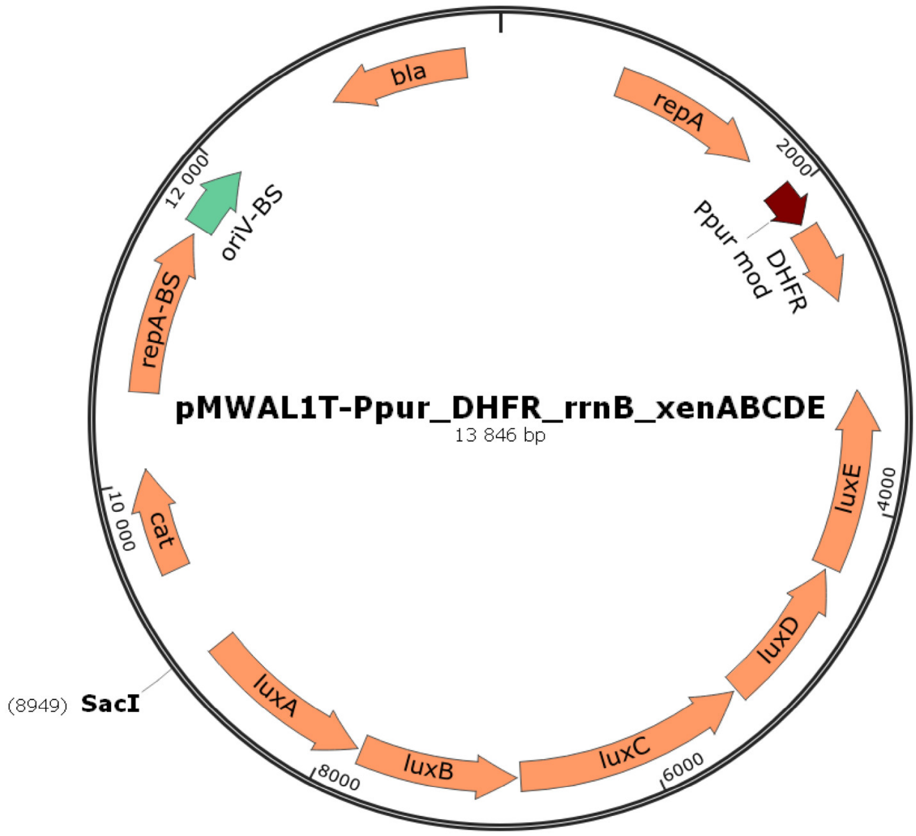

Figure S1: The map of hybrid plasmid pPL_ABCDExen [37]. Plasmid pPL_ABCDExen is romotorless shuttle vector with ewo replication origins from pMW118 and pBS72. The reporter genes are *luxABCDE* from *Photorhabdus luminescense*. The order of genes in the *lux*-operon and RBS upstream of each gene are optimized for *B. subtilis* expression. Resistance to trimethoprim (Tp^r), chloramphenicol (Cm^r), and ampicillin (Ap^r).

Table S1. List of primers used in the study. Italicized sequences is complementary to pPL_ABCDExen around the SacI site and were used for Gibson assembly.

alkAdir	*TAAAGAAGAGCTTTCAGGAATTCG*TTAAATAATTATAAGAAAACTCACGCTGG
alkArev	*GGCCGCGGTACCGAGCT*TGTAATAGCAAGATAACAAAATGAGTAAA
mrgADir mrgADir	*TAAAGAAGAGCTTTCAGGAATTCG*TTCCGATCGCTTTTTCCTTG
mrgARev	*GGCCGCGGTACCGAGCT*GATCTGTTGACTTAATTATATCATATACT
dinCDi	*GCCGCGGTACCGAGCT*TAATTACATTAAAGCAAACATA
dinCRev	*G TAAAGAAGAGCTTTCAGGAATTCG*AAACAGAACAAGTGTTCTTTTTT
promrev	CTGTCCCATGTCATTTCCTCC
promdir	ATTTCATAGAGAGTCCTCCTCTTGCTT

The sequences of promoter regions used for constructing new biosensor plasmids by insertion into SacI site of pPL_ABCDExen are given below:

- *The mrgA gene promoter, which is inducible by oxidative stress*

133 bp fragment of *B. subtilis* 168 gDNA

ttccgatcgctttttccttggtctgcgtgggagtctatcctgaagaaaaagctattcagctgatctaaattataattattataatttagtattgatttttatttagtatatg atataattaagtcaacagatc

- *The dinC gene promoter, which is inducible by DNA damages*

62 bp fragment of *B. subtilis* 168 gDNA

aaacagaacaagtgttctttttctattgaataccgaacgtatgtttgctttaatgtaatta

- *The alkA gene promoter,which is inducible by DNA alkylation*

85 bp fragment of *B. subtilis* 168 gDNA

tgtaatagcaagataacaaaatgagtaaagatgattatgtgataaactaatttcaaccagcgtgagttttcttataattatttaa
